# Impact of evolving strategies for arteriovenous graft creation and management on patency outcomes

**DOI:** 10.1080/0886022X.2025.2549776

**Published:** 2025-09-02

**Authors:** Yuan Luo, Xueqin Bian, Chunfeng Gu, Xian Wu, Linnan Bai, Jianbo Qing, Hong Ye, Chunsun Dai

**Affiliations:** ^a^ Kidney Diseases Center, The Second Affiliated Hospital of Nanjing Medical University, Nanjing, China; ^b^ Department of Nephrology, Zhejiang University Medical College Affiliated Sir Run Run Shaw Hospital, Hangzhou, China

**Keywords:** Arteriovenous graft, hemodialysis, patency rate, risk factors

## Abstract

**Background:**

Arteriovenous grafts (AVGs) are essential alternatives for hemodialysis patients who are unsuitable for arteriovenous fistulas (AVFs). As surgical expertise and monitoring strategies evolve, understanding the long-term performance and influencing factors of AVGs is crucial.

**Methods:**

This retrospective study analyzed 980 patients who underwent AVG creation at a single center between 2014 and 2022. Patients were grouped into three periods based on practice evolution. Primary and secondary patency rates at 6, 12, and 24 months were assessed via Kaplan–Meier’s analysis, and Cox regression was used to identify independent predictors of secondary patency.

**Results:**

Nine hundred and eighty patients were included (period I: 110; period II: 379; period III: 491). Over time, significant changes were noted in graft type selection (standard to Intering), anastomosis site (from antecubital to basilic vein), and adoption of endovascular interventions. Secondary patency rates significantly improved from period I to III (log-rank *p* < 0.001). Cox regression analysis identified risk factors for standard grafts, antecubital and brachial vein anastomosis, intervention count, and thrombosis.

**Conclusions:**

Over nine years, procedural refinements and enhanced surveillance strategies have significantly improved AVG outcomes, particularly secondary patency. These findings support the growing role of AVGs as a reliable and sustainable vascular access option for hemodialysis patients.

## Introduction

Hemodialysis remains the most widely utilized modality of kidney replacement therapy for patients with end-stage renal disease (ESRD) globally [1]. Effective vascular access (VA) management is a cornerstone of comprehensive care in maintenance hemodialysis (MHD). The arteriovenous fistula (AVF) is generally favored among the available options due to its superior patency and lower complication rates [2]. However, in patients with poor peripheral vasculature – often elderly individuals or those with diabetes and cardiovascular comorbidities – the feasibility of AVF creation is frequently limited [3–6]. Consequently, prosthetic arteriovenous grafts (AVGs) serve as a critical alternative.

In this growing and aging ESRD population, the prevalence of AVF non-maturation is increasing. AVF creation and maintenance often entail multiple interventions, prolonged catheter dependency, and higher healthcare costs than AVGs [7]. Given these factors, the secondary patency rates of AVGs may be comparable to those of AVFs [8–10].

In recent years, our center has observed a notable increase in AVG placements, a trend aligned with improved life expectancy among MHD patients and a growing recognition of AVGs as a viable long-term VA option [11,12]. Despite multiple technical strategies in AVG creation, including anastomosis site selection, graft material choice, and implantation location, comparative research on the impact of these variables, particularly regarding the use of supported versus non-supported grafts, remains limited [13–15].

The rise of endovascular techniques and increased vigilance in VA monitoring has further influenced practice patterns. However, their long-term effects on AVG patency outcomes are not yet well-defined. As one of the earliest centers in China to pioneer and refine AVG implementation, we have dedicated the past decade to developing individualized VA protocols guided by the evolving Kidney Disease Outcomes Quality Initiative (KDOQI) recommendations [16].

This study aims to evaluate the evolution of AVG-related strategies at our center from 2014 to 2023, specifically focusing on procedural selection and surveillance methods and their effects on primary and secondary patency outcomes.

## Method

### Study design and participants

This retrospective observational study included patients who underwent AVG creation at the Kidney Disease Center of the Second Affiliated Hospital of Nanjing Medical University between October 2014 and December 2022. Eligible patients were aged ≥18 years, had a confirmed diagnosis of ESRD, and received AVG creation using expanded polytetrafluoroethylene (ePTFE) grafts. Exclusion criteria were incomplete clinical data, follow-up duration shorter than one month, or a change in renal replacement therapy modality during follow-up.

Patients were categorized into three time periods based on institutional practice evolution:

*Period I* (October 2014–December 2016): Initial phase of AVG practice;
*Period II* (January 2017–December 2019): Advancement in surgical expertise;
*Period III* (January 2020–December 2022): Adoption of lower-limb AVG creation.

**Figure 1.**
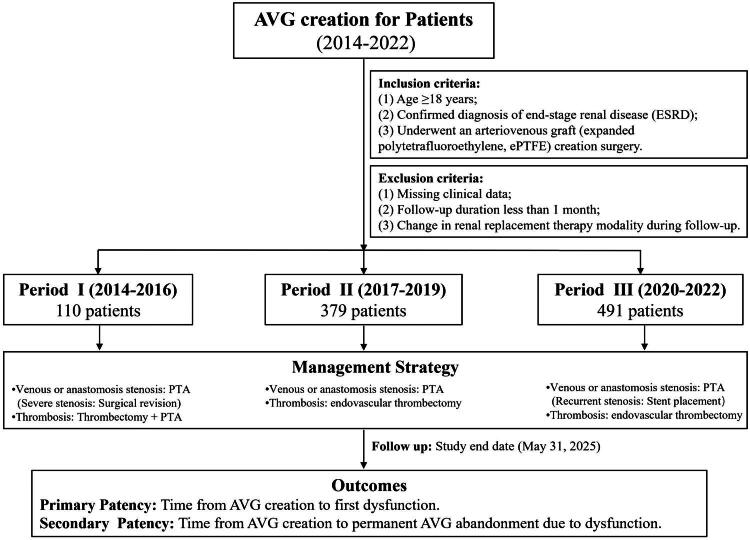
Flow diagram of this study. The flowchart depicts the patient enrollment process, the application of inclusion and exclusion criteria, the follow-up schedule, and the definitions of primary and secondary patency in the study cohort. Patients were initially assessed one month after AVG creation and subsequently followed every three months. Follow-up was terminated upon AVG abandonment, patient death, kidney transplantation, loss to follow-up, or at the study end date (31 May 2025). Primary patency was the time from AVG creation to the first dysfunction episode. Secondary patency was defined as the time from AVG creation to permanent graft abandonment due to dysfunction.

Demographic characteristics, comorbidities, history of previous VA, preoperative imaging, and surgical records were extracted from electronic medical records. The flow diagram of this study is shown in Figure 1.

### Creation strategy and preoperative evaluation

The institutional policy prioritized autologous VA and central vein preservation. Non-dominant limbs were preferred for AVG placement. Three types of grafts were employed: internally supported (Intering), standard (Standard), and early-cannulation (Acuseal), with the operating physician selecting the graft.

Preoperative evaluation included physical examination and duplex ultrasound (DU) mapping of the limb vasculature from the wrist to the axilla. Superficial and deep veins were assessed for patency, continuity, stenosis, and sclerosis. Patients with a history of central venous catheter (CVC) underwent angiography to assess central venous patency [16,17]. All procedures were performed by three experienced physicians in our center.

### Definition of AVG dysfunction

AVG dysfunction is categorized as follows: (1) *Stenosis*: defined as ≥50% luminal narrowing compared to adjacent segments, accompanied by at least one of the following: abnormal physical findings, blood flow <600 mL/min, elevated venous pressure, or prolonged bleeding post-dialysis [16,18]. (2) *Thrombosis*: Confirmed by the absence of a thrill or bruit during the physical examination and/or through imaging confirmation [19]. (3) *Other failure causes*: Including infection, pseudoaneurysm, seroma, dialysis access-related steal syndrome, and central venous stenosis [20].

### Follow-up and outcomes

Patients were initially evaluated one month after the AVG was created, and follow-ups were scheduled every three months. Follow-up was terminated at AVG abandonment due to dysfunction, patient death, loss to follow-up, or study end date (31 May 2025). The primary patency is the time from AVG creation to the first dysfunction episode. The secondary patency endpoint was the time until the AVG was permanently abandoned due to dysfunction [16,17].

### AVG monitoring and management

Throughout the past 9 years, our strategies for monitoring and managing AVGs have evolved significantly. In period I, monitoring and clinical physical exams were based on hemodynamic parameters during dialysis. Treatment for AVG thrombosis included thrombectomy combined with endovascular angioplasty, while severe venous anastomosis stenosis was treated through re-anastomosis. In period II, angiography was performed before AVG use, and patients were instructed to have ultrasound monitoring every three months. The management of thrombosis evolved toward endovascular thrombectomy, and endovascular angioplasty became the primary method for venous stenosis. In period III, residual or recurrent stenosis at the venous anastomosis was treated with stent placement. The KDOQI Clinical Practice Guidelines for Vascular Access guided our clinical practice. In earlier years, protocols were based on the 2006 edition [21]. Following the publication of the 2019 KDOQI guidelines, we adopted the updated recommendations, particularly those regarding AV access flow dysfunction, confirmation, and treatment [16]. Our management strategies, therefore, reflect the evolving standards and best available evidence as outlined in both guideline editions.

### Statistical analysis

All statistical analyses were performed using SPSS version 27.0 (IBM Corp., Armonk, NY) and R version 4.3 (R Development Core Team, Vienna, Austria). Continuous variables were summarized as means with standard deviations or medians with interquartile ranges, depending on data distribution assessed by the Kolmogorov–Smirnov test. Categorical variables were presented as frequencies and percentages. Group comparisons were conducted using one-way ANOVA for continuous variables and chi-squared tests for categorical variables, with *p* < 0.05 considered statistically significant.

Kaplan–Meier’s survival curves and log-rank tests were used to compare primary and secondary patency rates, with *p* < 0.05 indicating statistical significance. Cox proportional hazards regression models were applied to identify factors associated with patency. Variables with *p* < 0.20 in univariate analysis and clinically relevant factors were included in the multivariate model. The proportional hazards assumption was tested using time-dependent covariates based on interactions with log-transformed time. To account for death as a competing event, Fine and Gray’s subdistribution hazard models were conducted in R. A *post hoc* power analysis was also performed to confirm the adequacy of the sample size for key comparisons.

## Results

### Baseline characteristics of patients undergoing AVG surgery

Nine hundred and eighty patients were included in the study, with 110, 379, and 491 patients from periods I, II, and III, respectively. The mean annual surgical volume was 36.7 cases in period I, increasing to 126.3 in period II, and further rising to 163.7 in period III. There were no statistically significant differences observed among the three periods regarding patient age (period I: 62.1 ± 15.0 years, period II: 59.8 ± 12.6 years, period III: 59.5 ± 12.1 years, *p* = 0.148) or sex distribution (female: 40.0% in period I, 48.3% in period II, and 47.5% in period III, *p* = 0.293). Regarding dialysis duration, the proportion of pre-dialysis patients was highest in period II (8.7%), whereas period III showed the highest proportion of patients on dialysis for less than 1 year (49.3%). The proportion of patients on dialysis between 1 and 5 years peaked in period I (30.0%). Furthermore, the highest proportion of patients undergoing dialysis for more than 5 years was observed in period III. These differences in dialysis duration distribution among the three periods were statistically significant (*p* < 0.001). Analysis of comorbidities revealed no significant differences across the periods in the prevalence of diabetes mellitus (*p* = 0.225), hypertension (*p* = 0.869), and cerebrovascular disease (*p* = 0.231). However, a significantly higher proportion of patients with cardiovascular disease was observed in period I compared to subsequent periods (*p* < 0.001). There were significant differences among periods concerning previous VA types (*p* < 0.001). The proportion of patients with a prior AVF was highest during period I (52.7%), whereas the proportion of patients with non-cuff catheter (NCC) access was highest in period III (Table 1).

**Table 1.** Baseline characteristics of patients undergoing AVG surgery at three periods.

	Period I (2014–2016)	Period II (2017–2019)	Period III (2020–2022)	*p* Value
Number of patients	110	379	491	
Annual surgical volume	36.7	126.3	163.7	
Age (years)	62.1 ± 15.0	59.8 ± 12.6	59.5 ± 12.1	0.148
Sex (female, %)	44 (40.0%)	183 (48.3%)	233 (47.5%)	0.293
Duration of dialysis (*n*, %)				*<0.001*
Non-dialysis	7 (6.4%)	33 (8.7%)	28 (5.7%)	
<1 year	53 (48.2%)	169 (44.6%)	242 (49.3%)	
1–5 years	33 (30.0%)	93 (24.5%)	87 (17.7%)	
>5 years	17 (15.5%)	84 (22.2%)	134 (27.3%)	
Pathology (*n*, %)				0.209
Glomerulonephritis	17 (15.5%)	65 (17.2%)	75 (15.3%)	
Diabetes	45 (40.9)	154 (40.6%)	167 (34.0%)	
Hypertension	5 (13.6%)	66 (17.4%)	88 (17.9%)	
Systemic lupus erythematosus	4 (3.6%)	12 (3.2%)	18 (3.7%)	
Polycystic kidney disease	10 (9.1%)	19 (5.0%)	27 (5.5%)	
Unknown	15 (13.6%)	51 (13.5%)	81 (16.5%)	
Others	4 (3.6%)	12 (3.2%)	35 (7.1%)	
Comorbidities (*n*, %)				
Diabetes	52 (47.3%)	176 (46.7%)	203 (41.3%)	0.225
Hypertension	87 (79.1%)	302 (79.7%)	397 (80.9%)	0.869
Heart disease	26 (23.6%)	85 (22.4%)	58 (11.8%)	*<0.001*
Cerebrovascular disease	27 (24.5%)	66 (17.4%)	90 (18.3%)	0.231
History of VA (*n*, %)				*<0.001*
Non	7 (6.4%)	33 (8.7%)	25 (5.1%)	
AVF	61 (55.4%)	162 (42.8%)	202 (41.2%)	
AVG	3 (2.7%)	11 (2.9%)	24 (4.9%)	
TCC	17 (15.5%)	39 (10.3%)	75 (15.3%)	
NCC	21 (19.1%)	121 (31.9%)	152 (31.0%)	
Artery cannulation	0 (0.0%)	7 (1.8%)	10 (2.0%)	
Peritoneal dialysis	1 (0.9%)	6 (1.6%)	2 (0.4%)	
AVG location (*n*, %)				*0.004*
Fore-arm	65 (59.1%)	280 (73.9%)	335 (68.2%)	
Upper-arm	45 (40.9%)	99 (26.1%)	150 (30.5%)	
Lower-limb	0 (0.0%)	0 (0.0%)	6 (1.2%)	
Type of graft (*n*, %)				*<0.001*
Intering	35 (31.8%)	266 (70.2%)	334 (68.0%)	
Standard	75 (68.2%)	95 (25.3%)	150 (30.5%)	
Acuseal	0 (0.0%)	17 (4.5%)	7 (1.4%)	
First cannulation time (days)	35.5 (27.0–49.0)	29.0 (23.0–41.0)	34.0 (29.0–42.0)	0.050

*p* < 0.05, showing significant differences between groups are indicated in italics. AVF: arteriovenous fistula; AVG: arteriovenous graft; TCC: tunneled-cuff catheter; NCC: non-cuff catheter.

### Changes in AVG creation strategy

In period II, the proportion of upper-arm AVGs decreased to 26.1%, while in period III, the establishment of lower-limb AVGs commenced, accounting for 1.2% of the cases. Standard grafts were predominant in period I (68.2%), whereas Interring grafts became more prevalent in periods II and III, constituting 73.2% and 68.2%, respectively. Early-cannulation grafts were introduced in periods II and III, representing 1.6% and 1.4% of cases, respectively (Table 1).

**Figure 2.**
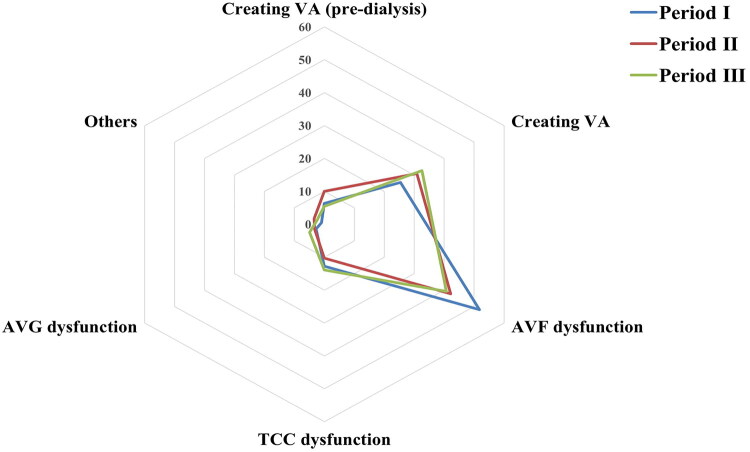
The indications for AVG creation during the three periods. Radar chart showing the distribution of clinical indications for arteriovenous graft (AVG) creation across period I (2014–2016), period II (2017–2019), and period III (2020–2022). Indications included AVF dysfunction, TCC dysfunction, AVG dysfunction, new access creation during pre-dialysis or dialysis (creating VA and creating VA pre-dialysis), and other causes. AVF dysfunction remained the most common indication in all periods, particularly in period I. Notably, pre-dialysis AVG creation was more common in period II, while AVG dysfunction increased slightly in period III.

Analysis of indications for AVG creation, including creating VA, creating VA (pre-dialysis), AVF dysfunction, AVG dysfunction, and other causes, revealed consistent trends across all three periods. AVF dysfunction was the primary indication in period I (51.8%), whereas creating VA (pre-dialysis) was relatively higher in period II (10.0%). AVG dysfunction was more frequently observed as an indication in period III (4.9%) compared to earlier periods (Figure 2).

**Figure 3.**
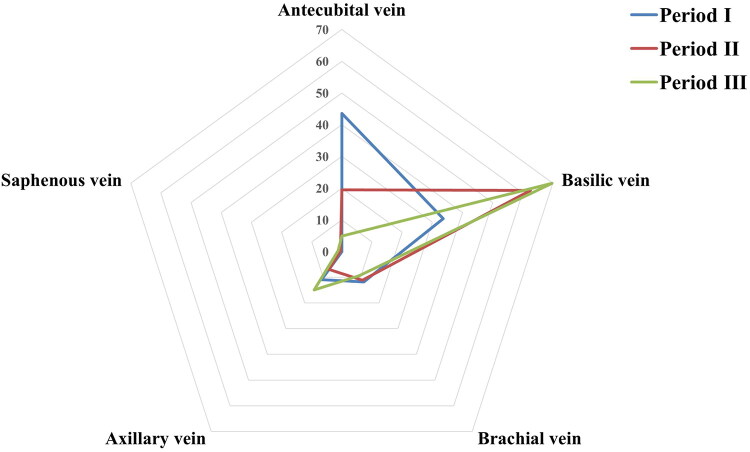
The choice of veins to anastomosis during the three periods. The radar chart demonstrates the distribution of vein types used for AVG anastomosis across period I (2014–2016), period II (2017–2019), and period III (2020–2022). A notable shift was observed from the antecubital vein in period I to a predominant use of the basilic vein in periods II and III. The brachial, axillary, and saphenous veins were infrequently used across all periods.

The selection of veins for anastomosis also varied significantly over the periods. During period I, the antecubital and basilic veins were predominantly utilized. However, in periods II and III, there was a clear preference for the basilic vein, irrespective of its location in the forearm or upper arm (Figure 3).

### Complications of AVGs

We assessed the incidence of complications, including stenosis, thrombosis, pseudoaneurysm, infection, and central vein stenosis. Compared with period I, the thrombosis was significantly lower in periods II and III (*p* < 0.05). Similarly, the pseudoaneurysm was also reduced in periods II and III compared to period I (*p* < 0.05). No significant differences were observed among the three periods regarding other complications (Table 2).

**Table 2.** Complications of AVGs.

	Period I (2014–2016)	Period II (2017–2019)	Period III (2020–2022)	
	Person-year	Person-year	Person-year	*p* Value
Stenosis	0.521	0.673	0.627	0.266
Thrombosis	0.455^a^^,^^b^	0.300^c^	0.296^c^	0.087
Pseudoaneurysm	0.059^a^^,^^b^	0.011^c^	0.008^c^	0.060
Infection	0.110	0.113	0.116	0.882
central vein stenosis	0.065	0.047	0.035	0.873
Total interventions	0.987	0.914	0.908	0.781

^a^ *p* < 0.05 compared to period II.

^b^ *p* < 0.05 compared to period III.

^c^ *p* < 0.05 compared to period I.

**Figure 4.**
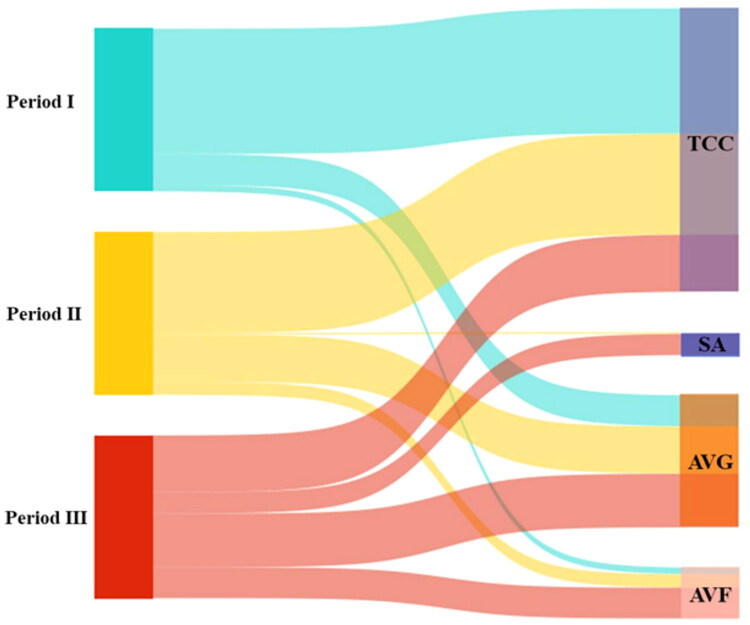
Vascular access selection after AVG failure over three periods. This Sankey diagram illustrates the flow of patients from period I (2014–2016), period II (2017–2019), and period III (2020–2022) to different types of vascular access following AVG failure. The access options include tunneled-cuff catheters (TCCs), arteriovenous grafts (AVGs), arteriovenous fistulas (AVFs), and superficialization arteries (SAs). Over time, there was a notable increase in the proportion of patients transitioning to AVGs and AVFs, with a corresponding reduction in long-term reliance on TCCs.

We also evaluated the subsequent VA strategies adopted by patients following AVG failure due to various causes. The four main approaches included tunneled-cuff catheter (TCC), AVG, AVF, and superficialization arteries (SAs). TCC was the most commonly chosen option. In some cases, TCC was used as a temporary access while managing complications such as infection or central vein stenosis, after which patients transitioned to another VA. However, the reliance on TCCs showed a downward trend over time, with a relative increase in the proportion of patients transitioning to either AVG or AVF creation in periods II and III. Only a small number of patients underwent SA procedures, primarily in the later periods (Figure 4).

### Primary and secondary patency of AVGs

**Figure 5.**
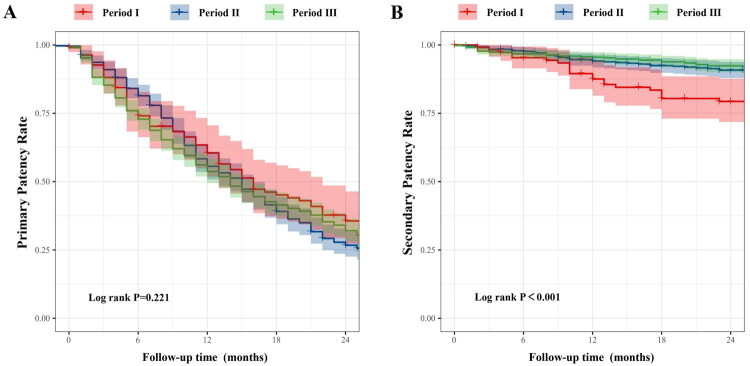
Twenty-four-month primary and secondary patency rates of arteriovenous grafts (AVGs) across the three study periods. (A) Kaplan–Meier’s curves showing primary patency over a 24-month follow-up for period I (2014–2016), period II (2017–2019), and period III (2020–2022). No significant difference was observed among the three periods (log-rank *p* = 0.221). (B) Kaplan–Meier’s curves for secondary patency over the same time frame revealed significantly higher patency rates in periods II and III compared to period I (log-rank *p* < 0.001).

**Figure 6.**
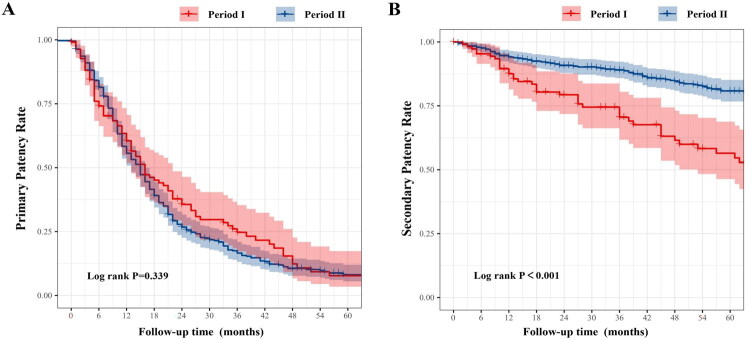
Sixty-month primary and secondary patency rates of AVGs comparing period I and period II. (A) Kaplan–Meier’s curves of primary patency showed no significant difference between periods I and II over a 60-month follow-up (log-rank *p* = 0.339). (B) Secondary patency was significantly improved in period II compared to period I throughout the 60 months (log-rank *p* < 0.001), indicating enhanced long-term outcomes with evolving graft management strategies.

Kaplan–Meier’s survival analysis demonstrated no statistically significant difference in primary patency rates among periods I, II, and III (log-rank *p* = 0.981) (Figure 5(A)). In period I, the primary patency rates at 6, 12, and 24 months were 63.6%, 54.5%, and 28.2%, respectively. In period II, the primary patency rates were 69.6%, 56.6%, and 26.2%, while in period III, they were 74.0%, 55.6%, and 27.8%, respectively. Regarding secondary patency, the 6-month rates were 90.0%, 94.4%, and 95.8% in periods I, II, and III, respectively. The 12-month rates were 88.5%, 92.1%, and 94.5%, and the 24-month rates were 77.5%, 90.3%, and 91.5%, respectively. Period I consistently exhibited the lowest secondary patency, while period III showed the highest (log-rank *p* < 0.001) (Figure 5(B)). Given that both periods I and II included follow-up durations exceeding 60 months, we further compared long-term patency outcomes between these two groups. The 36-month primary patency rates were 22.0% for period I and 13.4% for period II, while the 60-month primary patency rates were 2.0% and 5.3%, respectively. For secondary patency, the 36-month rates were 67.6% in period I and 90.3% in period II, and the 60-month rates were 26.5% and 66.3%, respectively. Kaplan–Meier’s survival analysis revealed no significant difference in 60-month primary patency rates between periods I and II (log-rank *p* = 0.339) (Figure 6(A)), whereas the 60-month secondary patency rate was significantly higher in period II compared to period I (log-rank *p* < 0.001) (Figure 6(B)).

Multivariate Cox proportional hazards regression analysis identified several independent predictors of AVG outcomes. Factors significantly associated with primary patency included hypertension (HR = 0.711, 95% CI: 0.566–0.894, *p* = 0.004), HDL <1.16 mmol/L (HR = 1.256, 95% CI: 1.036–1.522, *p* = 0.020), thrombin time (TT) <17.5 s (HR = 1.232, 95% CI: 1.022–1.486, *p* = 0.029), and use of the antecubital vein for anastomosis (vs. axillary vein; HR = 1.448, 95% CI: 1.071–1.959, *p* = 0.016) (Supplementary Table 1). Regarding secondary patency, independent predictors included treatment period (period II vs. I: HR = 0.635, 95% CI: 0.425–0.946, *p* = 0.026; period III vs. I: HR = 0.446, 95% CI: 0.284–0.702, *p* < 0.001), graft type (Standard vs. Interring-type; HR = 1.532, 95% CI: 1.118–2.100, *p* = 0.008), vein type (antecubital vs. axillary: HR = 2.286, 95% CI: 1.116–4.368, *p* = 0.024; brachial vs. axillary: HR = 2.617, 95% CI: 1.266–5.409, *p* = 0.009), interventions times (HR = 0.853, 95% CI: 0.808–0.899, *p* < 0.001), and occurrence of thrombosis (HR = 2.986, 95% CI: 2.089–4.268, *p* < 0.001) (Table 3).

**Table 3.** Multivariate Cox proportional hazards model analysis for secondary patency rates of AVG.

	HR	95% CI	*p* Value
Age			
≤65 years	Ref.		
>65 years	1.095	0.800–1.500	0.571
Sex			
Male	Ref.		
Female	1.211	0.894–1.641	0.217
Diabetes	1.256	0.810–1.733	0.166
Hypertension	0.948	0.643–1.398	0.788
Period			
I (2014–2016)	Ref.		
II (2017–2019)	0.635	0.425–0.946	*0.026*
III (2020–2022)	0.446	0.284–0.702	*<0.001*
Type of graft			
Intering	Ref.		
Standard	1.532	1.118–2.100	*0.008*
Vein to anastomosis			
Axillary vein	Ref.		
Cephalic vein	1.858	0.847–3.964	0.058
Basilic vein	1.833	0.979–3.524	0.124
Antecubital vein	2.286	1.116–4.684	*0.024*
Brachial vein	2.617	1.266–5.409	*0.009*
Intervention times	0.853	0.808–0.899	*<0.001*
Thrombosis			
No	Ref.		
Yes	2.986	2.089–4.268	*<0.001*

Due to the small number of patients with graft types such as Acuseal and anastomosed veins like the saphenous vein, they were not included in the analysis.

## Discussion

Over the past few years, the global prevalence of patients requiring hemodialysis for ESRD has steadily increased, resulting in a growing need for VA surgeries [22]. In the context of an aging dialysis population, rising diabetes incidence, and extended patient survival, AVGs have emerged as a valuable alternative for patients ineligible for AVF creation due to unfavorable vascular anatomy or comorbidities [23].

Our study, encompassing 980 cases over three defined periods, revealed that the strategies for AVG creation, monitoring, and management evolved considerably over time, contributing to significantly improved long-term outcomes, particularly in periods II and III. Analyzing our institutional experience, we identified multiple clinical and procedural factors influencing primary and secondary patency rates. These insights are critical for refining VA protocols in hemodialysis patients and supporting informed clinical decision-making.

The increasing annual surgical volume across the periods reflects the growing reliance on AVGs. In periods II and III, over 45% of patients had been on dialysis for less than one year, with approximately one-third having previously undergone AVF surgery. High AVF failure rates contribute to prolonged reliance on CVCs, which are associated with increased infection risk and healthcare costs [24,25]. Therefore, AVGs represent a practical and effective initial VA option for patients whose AVF creation is not feasible due to vascular or clinical limitations.

Notable changes were observed across the three periods in the indications for AVG creation, selection of anastomotic veins, and types of grafts utilized. While AVF failure or non-maturation remained the leading indication for AVG placement, there was a growing emphasis on early AVG creation in patients dependent on CVCs. This approach aims to reduce catheter dwell time and associated complications. The choice of anastomosis veins and graft types also shifted notably. Notable changes were observed across the three periods in the indications for AVG creation, selection of anastomotic veins, and types of grafts utilized. While AVF failure or non-maturation remained the leading indication for AVG placement, there was a growing emphasis on early AVG creation in patients dependent on CVCs. This approach aims to reduce catheter dwell time and associated complications. The choice of anastomosis veins and graft types also shifted notably. The cephalic vein was most commonly used in period I, whereas in periods II and III, the basilic vein became the preferred choice. The proportion of brachial vein anastomoses remained consistently low. These trends correlate with findings showing that the brachial vein has a lower secondary patency rate than the axillary vein. In period I, standard grafts were the most common, but intervening grafts became increasingly prevalent in the following periods. At the same time, the indications for AVG creation showed no significant changes across the three periods. These procedural adaptations reflect a learning curve shaped by accumulated surgical experience and evolving clinical evidence.

The primary causes of AVG dysfunction are stenosis and/or thrombosis resulting from neointimal hyperplasia and intraluminal narrowing [26,27]. Our analysis demonstrated that thrombosis rates were significantly higher in period I compared to later periods. This trend may reflect increased awareness of VA surveillance and advancements in endovascular technologies that facilitate timely detection and intervention for stenotic lesions, thereby preventing thrombotic complications. Additionally, period I exhibited the highest incidence of pseudoaneurysms and infections, particularly at cannulation sites – issues strongly linked to the quality of nursing care during AVG use [28]. In the early years, limited knowledge and clinical experience in downtown hospitals posed challenges for effective AVG management. However, with the implementation of targeted nursing education programs and adherence to standardized cannulation protocols, complication rates have since declined markedly.

AVG dysfunction has a direct impact on both primary and secondary patency. Starting from period II, our management strategies for stenosis and thrombosis shifted significantly toward endovascular approaches. Endovascular thrombectomy became the mainstay of treatment for thrombotic occlusion, while stent placement was increasingly adopted in period III to address residual or recurrent stenosis. Despite no significant differences in primary patency rates across the three periods, secondary patency rates improved notably in periods II and III. These findings suggest that advancements in endovascular therapies have played a crucial role in extending AVG lifespan.

Our observation of improved secondary patency without a corresponding increase in primary patency aligns closely with existing literature. While interventions such as endovascular thrombectomy and stent placement effectively restore AVG function after initial dysfunction, they inherently do not delay the initial occurrence of stenosis or thrombosis. Previous reports support this notion, suggesting that the primary determinants of initial patency involve biological factors and initial hemodynamic conditions rather than intervention timing alone [29,30]. Consequently, secondary patency, significantly influenced by timely detection and treatment of dysfunction, shows marked improvement with advanced endovascular strategies. This underscores the critical role of routine surveillance and early endovascular interventions, which can effectively prolong AVG survival and reduce catheter dependence, despite limited effects on the primary patency interval [15,28,29]. It should be noted that all analyses of primary and secondary patency in this study excluded lower-limb AVGs (limb-AVGs) to ensure the homogeneity and comparability of the study population.

Previous studies report 1- and 2-year primary patency rates of approximately 41% and 28% and secondary patency rates of 70% and 54%, respectively [28]. Chinese data indicate that the 1-, 2-, and 3-year primary patency rates range from 51.0 to 61.5%, 30.7 to 36.6%, and 15.4 to 23.2%, while secondary patency rates range from 85.6 to 96.7%, 68.6 to 90.1%, and 55.8 to 78.5%, respectively [29]. Our findings in periods II and III are consistent with these results, demonstrating secondary patency outcomes approaching those of AVFs.

In addition, AVGs offer the advantage of shorter maturation time, typically around 30 days, allowing earlier cannulation than AVFs, particularly in cases where AVF maturation is delayed or requires adjunctive procedures. This accelerated usability helps reduce or eliminate the need for prolonged CVC use, thereby preserving central venous resources and reducing catheter-related complications.

Our study also identified factors affecting primary and secondary AVG patency. We found that internally supported (Intering) grafts exhibit superior primary and secondary patency compared to unsupported (Standard) grafts, aligning with previous studies. Among the commonly used polytetrafluoroethylene (PTFE) grafts – internally supported, standard, and early-cannulation types – our analysis focused on Intering and standard grafts due to the limited number of Acuseal cases. The results suggest that standard grafts are associated with a significantly higher risk of patency loss. Improper post-cannulation handling or excessive external compression, particularly in patients experiencing post-dialysis hypotension, may contribute to thrombosis. In contrast, Intering grafts provide enhanced structural support that helps mitigate such risks [15]. We also found that the frequency of required interventions and the occurrence of thrombosis were significant predictors of secondary patency. Regular monitoring and timely endovascular management are essential for maintaining graft function, as more frequent interventions and the absence of thrombotic events were associated with improved long-term outcomes.

This study has several limitations. First, it was a single-center, retrospective analysis, which may introduce selection bias and limit the generalizability of our findings. Second, despite our efforts to ensure consistent follow-up durations across groups, some differences remain. Finally, improvements in patency outcomes may also be influenced by evolving operator experience, refinements in cannulation technique, dialysis technology upgrades, and overall advancements in patient care. Although not directly measurable in this retrospective design, these factors likely contributed to the enhanced results observed in later periods.

In conclusion, our experience over the past nine years demonstrates that continuous refinement in AVG creation, surveillance, and salvage techniques has improved long-term outcomes. With modern surveillance and intervention strategies, AVGs can achieve long-term outcomes, making them a suitable and practical option for VA in contemporary hemodialysis practice.

## Supplementary Material

Supplement table1 .docx
