# Supplementary material for: Impact of evolving strategies for arteriovenous graft creation and management on patency outcomes
Source: Ren Fail. 2025 Sep 2;47(1):2549776. doi: 10.1080/0886022X.2025.2549776 (PMC12409867; doi:10.1080/0886022X.2025.2549776)
Supplement: Supplement table1 .docx [file IRNF_A_2549776_SM7872.docx]

Supplement table 1. Multivariate Cox proportional hazards model analysis for patency rates of AVG.

|  | HR | 95%CI | P value |
| --- | --- | --- | --- |
| **Age** |  |  |  |
| ≤65y | Ref. |  |  |
| >65y | 0.919 | 0.752~1.124 | 0.410 |
| **Gender** |  |  |  |
| Male | Ref. |  |  |
| Female | 0.979 | 0.812~1.181 | 0.979 |
| **Diabetes** | 1.008 | 0.824~1.233 | 0.938 |
| **Hypertension** | 0.723 | 0.588~0.919 | *0.008* |
| **Period** |  |  |  |
| Ⅰ（2014~2016） | Ref. |  |  |
| Ⅱ（2017~2019） | 1.167 | 0.823~1.655 | 0.385 |
| Ⅲ（2020~2022） | 1.655 | 0.756~1.548 | 0.669 |
| **Hb(g/L)** |  |  |  |
| ＜110 | Ref. |  |  |
| ≥110 | 1.173 | 0.944~1.459 | 0.150 |
| **Alb(g/L)** |  |  |  |
| ＜35 | Ref. |  |  |
| ≥35 | 1.104 | 0.864~1.410 | 0.429 |
| **HDL-C(mmol/L)** |  |  |  |
| ≥1.16 | Ref. |  |  |
| ＜1.16 | 1.273 | 1.037~1.563 | *0.021* |
| **Ca(mmol/L)** |  |  |  |
| ＜2.2 | Ref. |  |  |
| ≥2.2 | 1.067 | 0.863~1.319 | 0.547 |
| **FER(ng/ml)** |  |  |  |
| ≥200 | Ref. |  |  |
| ＜200 | 1.233 | 1.013~1.500 | *0.037* |
| **TT(s)** |  |  |  |
| ≥17.5 | Ref. |  |  |
| ＜17.5 | 1.225 | 1.003~1.496 | *0.046* |
| **PT(s)** |  |  |  |
| ≥13.5 | Ref. |  |  |
| ＜13.5 | 1.392 | 0.974~1.990 | 0.069 |
| **FIB(g/L)** |  |  |  |
| ≥5.0  ＜13.5 | Ref. |  |  |
| ＜5.0 | 1.160 | 0.920~1.463 | 0.211 |
| **Type of graft** |  |  |  |
| Intering | Ref. |  |  |
| Standard | 1.287 | 1.044~1.586 | *0.018* |
| **Vein to anastomosis** |  |  |  |
| Basilic vein | Ref. |  | 0.050 |
| Cephalic vein | 0.951 | 0.676~1.337 | 0.772 |
| Antecubital vein | 1.043 | 0.647~1.683 | 0.862 |
| Brachial vein | 1.529 | 0.995~2.350 | 0.053 |
| Axillary vein | 1.171 | 0.768~1.784 | 0.463 |

Note: Due to the small number of patients with graft types such as Acuseal and anastomosed veins like the saphenous vein, they were not included in the analysis.
